# Supplementary material for: STAT5B leukemic mutations, altering SH2 tyrosine 665, have opposing impacts on immune gene programs
Source: Life Sci Alliance. 2025 Apr 14;8(7):e202503222. doi: 10.26508/lsa.202503222 (PMC11999048; doi:10.26508/lsa.202503222)
Supplement: Supplementary file 1 [file LSA-2025-03222_TableS1.docx]

**Supplementary Table S1.** Diagnoses of blood cancers in patients carrying STAT5B^Y665F^ or STAT5B^Y665H^ mutations.

| **Database** | **Munich Leukemia Laboratory (MLL)** | | | **Catalogue of Somatic Mutations in Cancer (COSMIC)** | |
| --- | --- | --- | --- | --- | --- |
| **Total cases surveyed** | 31,698 | | | 67,734 | |
| **STAT5B^Y665F^** | **Total blood cancer cases** | 53 (0.167%) | | **Total blood cancer cases** | 12 (0.018%) |
| **Diagnoses** | Mature T-cell neoplasm (unspecified) | 20 | | Unspecified | 12 |
|  | T-prolymphocyte leukemia  (T-PLL) | 9 | |  |  |
|  | T-cell/natural killer cell-large granular lymphocytic leukemia (T/NK-LGL leukemia) | 2 | |  |  |
|  | Hepatosplenic T-cell Lymphoma | 1 | |  |  |
|  | Lymphoma (unspecified) | 1 | |  |  |
|  | Chronic myelomonocytic leukemia (CMML) -1 | 1 | |  |  |
|  | Myelodysplastic/myeloproliferative with ring sideroblasts and thrombocytosis  (MDS/MPN RARS-T) | 1 | |  |  |
|  | Multiple myeloma | 1 | |  |  |
|  | Mature B-cell neoplasm (unspecified) | 1 | |  |  |
|  | Unspecified | 17 | |  |  |
| **STAT5B^Y665H^** |  | 0 |  | | 0 |
