# Supplementary material for: STAT5B leukemic mutations, altering SH2 tyrosine 665, have opposing impacts on immune gene programs
Source: Life Sci Alliance. 2025 Apr 14;8(7):e202503222. doi: 10.26508/lsa.202503222 (PMC11999048; doi:10.26508/lsa.202503222)
Supplement: Supplementary file 6 [file LSA-2025-03222_TableS6.docx]

**Supplementary Table S6.** Sequences of sgRNA for CRISPR/Cas9 and base-editing targeted mice. The donor oligo is contained the desired Y (TAC) to F (TTT) change.

| Target site | sgRNA sequences |
| --- | --- |
| STAT5B^Y665H^ | 5’-TGAGGTAATTCAGGTCCCCCAGG-3’ |
| STAT5B^Y665F^ | 5’-TGAGGTAATTCAGGTCCCCCAGG-3’  Donor Oligos  GGAATCTGATGCCTTTTACCACTAGAGACTTCTCTATCCGGTCCCTCGCTGA  CCGCCTGGGGGACCTGAATTTTCTCATATATGTGTTTCCTGATCGGCCAAAG  GATGAAGTATATTCTAAGTACTACACACC |
